# Single-cell RNA sequencing advances in revealing the development and progression of MASH: the identifications and interactions of non-parenchymal cells

**DOI:** 10.3389/fmolb.2025.1513993

**Published:** 2025-03-25

**Authors:** Meng Ning, Donghui Lu, Dong Liang, Pei-Gen Ren

**Affiliations:** ^1^ Department of Endocrinology, Peking University Shenzhen Hospital, Shenzhen, China; ^2^ Center for Energy Metabolism and Reproduction, Shenzhen Institute of Advanced Technology, Chinese Academy of Sciences, Shenzhen, China; ^3^ Department of Endocrinology, First Affiliated Hospital of Harbin Medical University, Harbin, China; ^4^ Center for Cancer Immunology, Shenzhen Institute of Advanced Technology, Chinese Academy of Sciences, Shenzhen, China; ^5^ University of Chinese Academy of Sciences, Beijing, China

**Keywords:** MASLD, MASH, single-cell sequencing, macrophages, hepatic sinusoidal endothelial, hepatic stellate cells, NASH

## Abstract

Developing drugs for the treatment of Metabolic Associated Steatohepatitis (MASH) has always been a significant challenge. Researchers have been dedicated to exploring drugs and therapeutic strategies to alleviate disease progression, but treatments remain limited. This is partly due to the complexity of the pathophysiological processes, and inadequate knowledge of the cellular and molecular mechanisms in MASH. Especially, the liver non-parenchymal cells (NPCs) like Kupffer cells, hepatic stellate cells and sinusoidal endothelial cells which play critical roles in live function, immune responses, fibrosis and disease progression. Deciphering how these cells function in MASH, would help understand the pathophysiological processes and find potential drug targets. In recent years, new technologies have been developed for single-cell transcriptomic sequencing, making cell-specific transcriptome profiling a reality in healthy and diseased livers. In this review, we discussed how the use of single-cell transcriptomic sequencing provided us with an in-depth understanding of the heterogeneous, cellular interactions among non-parenchymal cells and tried to highlight recent discoveries in MASH by this technology. It is hoped that the summarized features and markers of various subclusters in this review could provide a technical reference for further experiments and a theoretical basis for clinical applications.

## 1 Introduction

**FIGURE 1:**
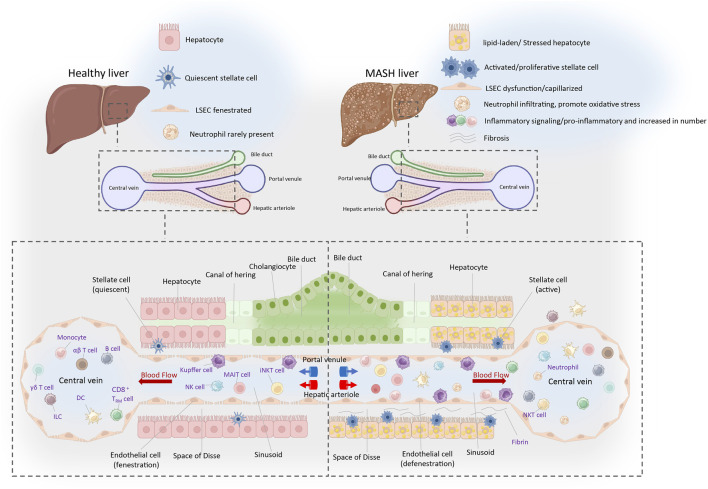
Immune landscape of the healthy and MASH liver. The portal triad, composing the hepatic artery, portal vein, and bile duct, constitutes a crucial anatomical structure amidst hepatic lobules. The liver receives a dual blood supply from both the portal vein and hepatic artery, with blood coursing through hepatic lobules from these vessels towards the central vein. The liver serves as a pivotal site for the immune cells, which circulate or transiently patrol within the hepatic sinusoids or the liver parenchyma. This is crucial for maintaining immune homeostasis locally and systemically. The resident cells mainly include Kupffer cells, CD8+tissue-resident memory T cells (CD8+TRM) and ILCs. Cells that circulate or temporarily patrol within the hepatic sinusoids or liver parenchyma include neutrophils, NK cells, monocytes, B cells, DCs, NKT cells, iNKT cells, MAIT cells, γδT cells, CD4+and CD8+αβT cells, and others. The space of Disse is an important site for material exchange between blood and hepatocytes. Nutrients, hormones, and waste products in the plasma pass through the pores of the liver sinusoidal endothelial cells (LSECs) into the space of Disse, where they are then absorbed or metabolized by hepatocytes. In the condition of MASH, the number of activated immune cells significantly increases. HSCs located in the space of Disse become activated and transformed into myofibroblasts, which synthesize and secrete substantial quantities of collagen and other components of the extracellular matrices, culminating in the development of liver fibrosis. Well-differentiated LSECs (fenestrae-Stabilin1/2, LYVE-1): Inhibit HSCs activation and promote hepatocyte proliferation (Zhang et al., 2020). Dedifferentiated LSECs (capillarization-CD31, CD34): Form a basement membrane, leading to HSCs activation and hepatocyte injury (Kumar et al., 2021; Gracia-Sancho et al., 2021). Abbreviations: NK, natural killer cell; DC, dendritic cell; iNKT, invariant natural killer T cell; MAIT, mucosal-associated invariant T cell; ILC, innate lymphoid cell.

Metabolic dysfunction-associated fatty liver disease (MASLD) is a prevalent chronic liver condition closely linked to metabolic syndromes such as obesity, type 2 diabetes, and hyperlipidemia. The progression of MASLD begins with metabolic dysfunction-associated steatotic liver (MASL) and can advance to MASH (Lekakis and Papatheodoridis, 2024). MASH may further progress to liver fibrosis, cirrhosis, and even hepatocellular carcinoma (HCC) (Diehl and Day, 2017). The global prevalence of MASLD is approximately 25%, rising to about 60% in obese individuals and 80% in patients with type 2 diabetes (Younossi et al., 2016). MASH constitutes about 20%–30% of MASLD cases and is a leading cause of liver disease-related mortality (Dulai et al., 2017). In the pathogenesis and progression of MASH, the aberrant activation and interaction of various hepatic cellular populations disrupt the homeostasis of the liver and the whole organism, culminating in a multifaceted pathological mechanism (Figure 1). Kupffer cells (KCs), the resident macrophages of the liver, are responsible for phagocytosing pathogens and cellular debris while playing a crucial role in liver immunity. In the context of MASH, KCs become activated and secrete substantial quantities of pro-inflammatory cytokines, such as Tumor Necrosis Factor-alpha (TNF-α) and Interleukin-1 beta (IL-1β). This exacerbates hepatic inflammation and contributes to hepatocellular damage. Liver sinusoidal endothelial cells (LSECs), specialized endothelial cells within the liver, possess a unique sieve-like structure that facilitates the regulation of material exchange and cell migration. In MASH, dysfunction of LSECs, such as defenestration (loss of fenestrae), can exacerbate liver inflammation and fibrosis. Hepatic stellate cells (HSCs) become activated upon liver injury, transforming into a myofibroblast-like phenotype and secreting large amounts of extracellular matrix proteins, which contribute to liver fibrosis. The activation of HSCs is triggered by signals from various cells, including hepatocytes, KCs, and LSECs. These three cell types communicate and interact, collectively forming a complex molecular signaling network that regulate the progression of MASH.

**FIGURE 2:**
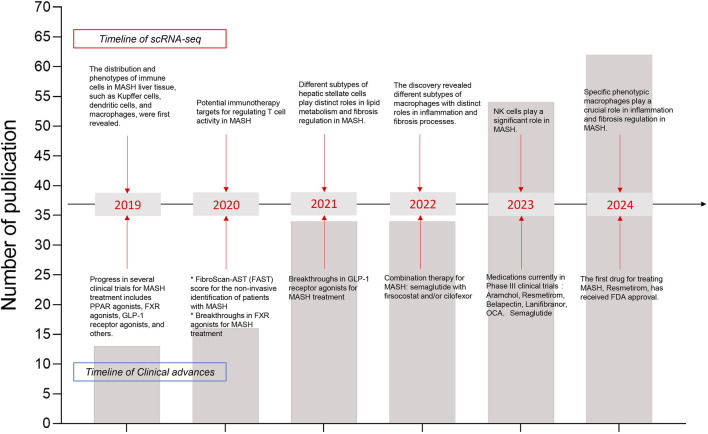
Timeline of recent years’ major research findings on MASH disease using scRNA-seq has been summarized. The line chart reflected the total number of publications. Arrows point to major discoveries that laid the foundation for our understanding of annual advancements in scRNA-seq (upper boxes) and clinical research progress (lower boxes). In recent years, the number of publications in single-cell transcriptomics has increased annually, which deepening our understanding of the pathophysiology of MASH.

In 2019, Dominic Grün’s team successfully utilized single-cell RNA sequencing (scRNA-seq) to construct a detailed map of cell populations and novel cell subtypes in the healthy human liver (Aizarani et al., 2019). This groundbreaking discovery provided a multidimensional perspective on both normal and diseased livers, significantly enhancing our understanding of liver development and function. Furthermore, scRNA-seq studies of the liver have proven invaluable for identifying potential targets for immunotherapy and clinical treatments. As the Frontier of genomics, scRNA-seq is developing rapidly. Recently, new discoveries and breakthroughs have emerged from scRNA-seq studies in both human and murine models of MASH (Povero et al., 2023; Rosenthal et al., 2021; Meng et al., 2024; Qing et al., 2022) (Figure 2). Notably, scRNA-seq has underscored the crucial roles of recruited monocyte-derived and bone marrow-derived macrophages in the progression of MASH (Peiseler et al., 2022). Additionally, scRNA-seq enables high-resolution transcriptomic profiling of mesenchymal subpopulations in liver fibrosis, revealing that the release of chemokines and cytokines, as well as extracellular matrix production, varies among hepatic stellate cells (HSCs) and myofibroblast subpopulations (Rosenthal et al., 2021; Dobie et al., 2019; Krenkel et al., 2019). A series of studies utilizing scRNA-seq has provided unprecedented insights into the heterogeneity of hepatic immune cells, revealing striking alterations in MASLD/MASH (Blériot et al., 2021; Daemen et al., 2021; Ramachandran et al., 2020; Remmerie et al., 2020; Seidman et al., 2020; Tran et al., 2020).

This review summarized the latest research findings, using scRNA-seq technology, on liver macrophages/KCs, LSECs, and HSCs in both healthy liver and MASH. These findings are expected to provide valuable references for future research, pharmaceutical development, and theoretical studies.

## 2 Kupffer cell/macrophage

### 2.1 Liver macrophage turnover in health and MASH

Hepatic macrophages have been proposed to comprise a number of cell subpopulations, forming a major innate defense system in liver. Among these subpopulations, KCs constitute 80%–90% of the total macrophage population, representing the predominant component of the reticuloendothelial system. KCs are embryonically derived macrophages that reside within the liver sinusoids and possess the capability of self-renewal and migration (Remmerie et al., 2020; Seidman et al., 2020; Krenkel et al., 2020; Xiong et al., 2019). Recent studies employing a broad set of microscopy techniques have demonstrated that KCs are not solely confined to the liver sinusoids but extend significant portions of their cellular structures into the perisinusoidal space of Disse. This anatomical arrangement facilitates intimate interactions with HSCs and hepatocytes, which are more complex than previously understood (Bonnardel et al., 2019). In healthy liver, KCs play pivotal roles in preserving liver function and immune system health. They achieve this by phagocytosing and degrading harmful substances in the blood, engaging in immune responses, and releasing cytokines to modulate inflammatory processes. Additionally, KCs are instrumental in maintaining hepatic homeostasis, ensuring the liver operates optimally and maintains resilience (Kumar et al., 2021). In the progression of MASH, hepatic lipid accumulation and oxidative stress induce KCs activation and functional overload. Sustained inflammatory stimuli contribute to KCs depletion and apoptosis. Dying KCs promptly release cytokines such as tumor necrosis factor and IL-1, which can activate HSCs and LSECs. Activated HSCs and LSECs orchestrate the recruitment and adhesion of monocytes by transiently secreting chemokines and adhesion molecules. The process forms a sophisticated intercellular signaling network that enhances monocyte infiltration and function (Bonnardel et al., 2019). The monocyte-derived macrophages (MoMF), once recruited to the liver, work alongside of KCs in the progression of MASH. They actively contribute to steatosis, inflammation, and fibrosis development, exacerbating liver damage. Advances in single-cell transcriptomics have revolutionized our understanding of macrophages and their subpopulations, enabling precise identification of pathogenic macrophage subsets. Current research is exploring targeted strategies aimed at specific macrophage subgroups, polarization states, and inflammatory responses as potential therapeutic approaches for managing MASH.

### 2.2 Macrophages heterogeneity and plasticity in the healthy and MASH liver

ScRNA-seq studies have unraveled the heterogeneity of macrophages within the murine liver, with different origins and subpopulations exhibiting distinct functions. Embryo-derived Kupffer cells (EmKCs) are divided into two subgroups: CD206^low^ESAM^−^ Kupffer cell population and the less abundant CD206^high^ESAM^+^ Kupffer cell population (Blériot et al., 2021; De Simone et al., 2021) (Table.1). In a healthy liver, few recruited macrophages are derived from bone marrow monocytes. These monocytes circulate within the liver sinusoids but do not integrate into the KC pool (Remmerie et al., 2020; Seidman et al., 2020; Scott et al., 2016) (Figure 1). In MASH conditions, the KC pool undergoes continuous changes. Lipid-induced endoplasmic reticulum (ER) stress leads to the progressive death of EmKCs, and recruited monocyte-derived Kupffer cells (MoKCs) gradually enter the KC pool. Meanwhile, both ER stress and MoKCs could impair the self-renewal of EmKCs (Remmerie et al., 2020; Seidman et al., 2020; Krenkel et al., 2020; Xiong et al., 2019; Wang and Gao, 2021). Compared to EmKCs, MoMF have a shorter residency period in the liver. These immature macrophages are crucial in promoting an inflammatory milieu. MoMF acquire mature EmKC markers following the resolution of lipid-induced inflammation (Table.1). When KCs are depleted, circulating monocytes are recruited to the liver and rapidly express lineage-determining transcription factors (LDTFs) specific to KCs, subsequently differentiating into KC-like cells that express a specific subset of KC genes. Transcriptionally, MoKCs are highly similar to EmKCs, and Clec4f^+^ macrophages are highly similar to EmKCs (Scott et al., 2016; van de Laar et al., 2016; Sakai et al., 2019). While MoKCs exhibit significant similarity to EmKCs, lipid-associated macrophages (LAMs) differ considerably from EmKCs, especially in lipid metabolism and immune activation (Remmerie et al., 2020) (Table.1).

**TABLE 1:** ScRNA-seq analysis of subsets markers of macrophages in the mouse liver under different condition (Xiong et al., 2019; Wang and Gao, 2021; Barreby et al., 2022).

Cell	Health mice				MASLD/MASH mice				MASLD/MASH mice	
Macrophages	Different Origins	Subsets	Cell Markers	Location	Different Origins	Subsets	Cell Markers	Location	Regression	
	Embryonic Kupffer cells (Clec4f+, Timd4+, Cd163+, Vsig4+)	KC1	Clec4f+ ,Timd4+ (Cd206low Esam–)	Located with the hepatic sinusoids	Embryonic Kupffer cells	KC1	Clec4f+ ,Timd4+ (Cd206low , Esam–)	Located with the hepatic sinusoids	Kupffer cell derived from monocytes (MoKC)	Clec4flow , Timd4low, Cd163-, Vsig4+
		KC2	Clec4f+ ,Timd4+ (Cd206high Esam+, Lyve1, Cd36)			KC2	Clec4f+ ,Timd4+ (Cd206high , Esam+)			
	Recruited liver macrophages		Clec4f– Timd4– (Adgre1+ Cx3cr1+ Ccr2+, Itgam+)	Preferentially localized in the portal areas	Macrophages derived from monocytes (MDM)	Transitional macrophages	Clec4f–, Timd4–, Clec2+ (Cx3cr1, Itgax, H2-M2, Olfml3)	Rapidly mobilised to sites		
						Recruited Kupffer cell (from transitional macrophages)-MoKCs	Clec4f+, Timd4– (Vsig4, C6, Clec4f, Cd207, Tgfb3)			
						CCR2-dependent lipid-associated macrophage	Clec4f–, Timd4– (Cx3cr1, Ccr2)	Accumulating pericentrally in steatotic livers		
						Lipid-associated macrophage (from CCR2-dependent lipid-associated macrophage)-LAMs	Clec4f–, Timd4– (Spp1, Cd9, Trem2, Gpnmb, Cd63)			
						Kupffer cell derived from monocytes (MoKCs)	Clec4flow , Timd4-, Cd163-, Vsig4low	Located with the hepatic sinusoids		

Inflammatory Ly-6C^high^ monocytes in mice correspond to human classical monocytes (CD14^+^CD16^low^). In contrast, mouse patrolling Ly6C^low^ monocytes correspond to human non-classical monocytes (CD14^low^CD16^+^) (Papachristoforou and Ramachandran, 2022). In mice, Clec4f is primarily used to label embryo-derived Kupffer cells (EmKCs) to distinguish and identify resident Kupffer cells in the liver, however, it is expressed only in the late developmental stages of KCs. CLEC2 is an early marker of KCs, continuously expressed throughout their lifecycle, making it one of the most valuable and earliest markers for selectively differentiating EmKCs from MoMF (Tran et al., 2020; Scott et al., 2016). Clec4f is not conserved in humans, and the most reliable protein marker for human KCs is VSIG4, identified through CITE-seq (Cellular Indexing of Transcriptomes and Epitopes by Sequencing) analysis (Guilliams et al., 2022). Notably, MoKCs can also express Clec4f, representing an adaptive mechanism of the liver in response to injury or disease states, partially compensating for the loss of EmKCs’ functions and thereby helping to restore and maintain liver immune function and homeostasis. EmKCs, being long-term resident macrophages, exhibit superior stability in both residency and regeneration capacity. Conversely, MoKCs generally function as transient responders, mobilized during acute or chronic inflammatory conditions. Moreover, MoKCs may differ from EmKCs in gene expression profiles, cellular metabolism, and functional characteristics. In summary, during the progression of MASLD/MASH, liver macrophages exhibit considerable heterogeneity, but the proportions of resident and recruited macrophages differ between mice and humans. In a steady state, in mice, liver macrophages are primarily EmKCs, whereas in humans, liver macrophages are predominantly replenished from circulating monocytes (Barreby et al., 2022). These studies substantiate the association between inflammation and macrophage recruitment in MASH, underscoring the considerable plasticity of liver macrophages. KCs, integral to liver function, dynamically adjust their functions and phenotypes in response to hepatocyte injury and lipid overload. During the initial phases of liver injury, KCs frequently transition to a pro-inflammatory phenotype, releasing inflammatory mediators in response to tissue damage. Subsequently, to mitigate and resolve inflammatory damage, the specific autocrine molecular signals, such as anti-inflammatory cytokines, would induce these macrophages to transition into a phenotype that facilitates tissue repair and resolution of fibrogenesis (Tacke, 2017). Overall, monocytes have three molecular expression patterns: the initial state (circulating monocytes), activation and functional differentiation state at the site of inflammation, and crossing the endothelial barrier. The molecular expression patterns at different stages reflect their specific functional roles in inflammation and tissue repair. The expression of specific molecules is crucial for monocytes to cross the endothelial barrier and migrate to the site of inflammation. Monocyte sequencing technology and dynamic tracking studies provide essential tools for understanding these processes. By regulating and targeting key molecules, it may be possible to “turn on” or “turn off” the inflammatory niche, thereby controlling monocyte recruitment and action to slow disease progression (Bonnardel et al., 2019).

Moreover, despite the sophisticated heterogeneity of macrophages in mouse and human MASH, certain gene regulations or pathway activations remain highly consistent: ECM signaling, the peroxisome proliferator-activated receptor (PPAR) pathway, and chemokines in the macrophage niche (Xiao et al., 2023; Li W. et al., 2023). Targeting these signaling pathways could provide alternative options for drug design, such as Galectin three inhibitors (which have been tested in clinical trials and were effective in reducing portal hypertension and fibrosis) (Al Attar et al., 2021), C-C chemokine receptor 2 (CCR2) and CCR5 inhibitors (which show positive effects on liver fibrosis, but are not effective at preventing the progression of hepatic steatosis) (Barreby et al., 2022), and PPAR agonists (Francque S. et al., 2021; Francque S. M. et al., 2021).

### 2.3 Both hepatic EmKCs and MoKCs promote MASH progression

**FIGURE 3:**
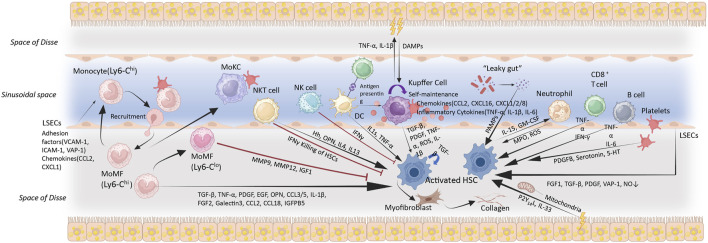
Immune cell activation, recruitment and cellular crosstalk in MASH progression. The accumulation of hepatic lipids and lipid metabolites constitutes a key pathological event in MASH. These alterations instigate a cascade of oxidative and organelle stress responses, which culminating in sublethal or lethal hepatocyte injury. Cytokines, chemokines, and DAMPs released by damaged hepatocytes activate and alter the function of non-parenchymal cells (including LSECs, Kupffer cells, HSCs, and other immune cells), eventually resulting in liver inflammation and fibrosis. Metabolic injury causes various cell types interactions in the liver via the production of hormones, cytokines, and other signalling molecules, which would result in HSC activation. These cells could promote (black arrows) or inhibit (red arrows) HSC activation through different mechanisms, thereby influence the progression of liver fibrosis. Abbreviations: VCAM-1, vascular cell adhesion molecule-1; ICAM-1, intercellular adhesion molecule-1; VAP-1, vascular adhesion protein 1; CCL, (C-C motif chemokine); CXCL, C-X-C motif chemokine; EGF, epidermal growth factor; FGF, fibroblast growth factor; IGFBP5, insulin-like growth factor binding protein 5; PDGF, platelet-derived growth factor; ROS, reactive oxygen species; TGF, transforming growth factor; TNF-α, tumour necrosis factor α; 5-HT, 5-hydroxytrypamine; MMP, matrix metalloproteinase; DAMPs/PAMPs, damage/pathogen-associated molecular patterns; MPO, myeloperoxidase; GM-CSF, Granulocyte-Macrophage Colony-Stimulating Factor; OPN, Osteopontin; HRG, Histidine-Rich Glycoprotein; MoMF, monocyte-derived macrophage; MoKC, monocyte-derived Kupffer cell; NO, nitric oxide; P2Y14L, P2Y14 ligands. MoMF (Ly6-C^high^): Monocytes generated in the bone marrow migrate through the bloodstream to the liver, where they would be differentiated. MoMF (Ly6-C^low^): Play roles in anti-inflammatory responses and tissue repair, regulating the immune response.

The roles of EmKCs and MoKCs in MASH-related inflammation and fibrosis remain debated. RNA sequencing of mouse liver macrophages has revealed that inflammatory markers are exclusively expressed in monocyte-derived macrophages during obesity and hepatic steatosis, whereas EmKCs do not exhibit these markers (Morinaga et al., 2015). Researchers found that the expression of MCP-1/C-C chemokine receptor 2 (CCR2) in MoKCs is five times higher than in EmKCs, and obese mice recruit more monocytes with a pro-inflammatory phenotype than lean mice (Morinaga et al., 2015). Most KCs are EmKCs, which are more conducive to triglyceride (TG) storage and help prevent excessive lipid accumulation and metabolic disorders (Tran et al., 2020). Conversely, MoKCs hinder hepatic TG storage and exhibit more pro-inflammatory and pro-fibrotic characteristics. These traits can aggravate liver damage in the context of obesity and MASH by amplifying inflammatory responses and promoting fibrosis (Blériot et al., 2021; Remmerie et al., 2020; Seidman et al., 2020; Tran et al., 2020; Krenkel et al., 2020; Xiong et al., 2019). However, some evidence suggests that MASH progression is driven by EmKCs rather than infiltrating monocytes (Tacke, 2017; Morinaga et al., 2015). EmKCs, when stimulated by lipids, pathogens, and other damage-associated signals, become activated and release large amounts of pro-inflammatory cytokines, chemokines, and reactive oxygen species, which can directly cause hepatocyte injury and apoptosis. They can also induce hepatocyte apoptosis via death receptor pathways such as Fas/FasL and TRAIL/DR5. Moreover, EmKCs secrete pro-fibrotic factors like TGF-β and PDGF, which activate HSCs and promote liver fibrosis (Karlmark et al., 2009; Mehal and Schuppan, 2015; Pellicoro et al., 2014; Ramachandran et al., 2012; Tacke and Zimmermann, 2014) (Figure 3).

Research has established that KCs mediate intrahepatic platelet recruitment from the early phases of MASLD to the advanced stages of MASH. Platelets contribute to liver inflammation and injury, by promoting immune cell recruitment during MASH. Platelets infiltrate the liver via hyaluronan-CD44 binding, a process dependent on the presence of KCs. This research underscores the important role of platelets, especially GPIbα, in the pathogenesis of MASH and its progression to hepatocellular carcinoma, proposing that antiplatelet therapy could be a promising treatment strategy (Malehmir et al., 2019). Both EmKCs and MoKCs likely participate in platelet recruitment. MoKCs exhibit significant pro-inflammatory and pro-fibrotic characteristics in MASH, EmKCs drive inflammation and fibrosis in response to lipid accumulation and damage signals. The intricacy of these multiple mechanisms highlights the multifactorial nature of liver disease progression, offering crucial insights for subsequent research and therapeutic interventions.

### 2.4 The role of macrophages in lipid metabolism

Hepatic lipotoxicity refers to the toxic effects of intermediate products generated during fatty acid metabolism on hepatocytes and is considered one of the core mechanisms driving the progression from MASLD to MASH. These lipotoxic intermediates, such as diacylglycerols and ceramides, directly damage hepatocytes, leading to apoptosis and necrosis. The damaged hepatocytes release damage-associated molecular patterns (DAMPs) that activate hepatic immune cells, thereby exacerbating inflammation. Lipotoxicity also directly promotes HSC activation by inducing the secretion of fibrotic factors like TGF-β. Fat-laden hepatocytes secrete cytokines and chemokines such as CCL2 and extracellular vesicles, which in turn activate non-parenchymal cells such as liver macrophages, HSCs, and LSECs (Rada et al., 2020).

ScRNA-seq data, combined with cell-cell interaction and ligand-receptor analyses, have elucidated the impact of lipotoxicity on interactions among hepatocytes, immune cells, and HSCs, thus driving inflammation and fibrosis progression (Ramachandran et al., 2019). The specific activation of KCs involves the coordinated actions of hepatocytes, HSCs, and LSECs. These cells express ligands such as TGFβ, CSF1, BMP9, DDL4, and desmosterol, which activate transcription factors in hepatic macrophages, initiating Kupffer cell-specific transcriptional programs (Bonnardel et al., 2019; Scott et al., 2016; Sakai et al., 2019).

Excessive free cholesterol is also a non-negligible factor causing hepatocytes lipo-toxic-death, can lead to upregulation of pro-fibrotic factors in hepatocytes and HSCs, triggering inflammation and ECM production in MASH (Gan et al., 2014; Wang et al., 2020). In both mice and humans with MASH, a unique liver tissue structure called the crown-like structure (CLS) has been observed. This architecture comprises CD11c^+^ macrophages which envelop and phagocytose dead or dying hepatocytes. These hepatocytes are mostly containing sizable lipid droplets, or accompanied with abundant cholesterol crystals (Itoh et al., 2017). Cholesterol overload in macrophages induces lysosomal stress, initiating fibrosis (Itoh et al., 2023).

Regulating fatty acid and cholesterol metabolism, along with macrophage function, represents key strategies for addressing MASH. Sc-RNA-seq technologies offer a novel perspective on elucidating the specific role of lipotoxicity in MASH progression, thereby identifying potential therapeutic targets. More techniques like single-cell ATAC-seq (chromatin accessibility analysis) utilized in diverse macrophages subpopulations during health and MASH could further illuminate how epigenomic diversity modulates macrophage functionality.

### 2.5 Periportal macrophages act as “sentinel” in MASH

Compromised intestinal barrier function facilitates the translocation of microbiota and their metabolites into the liver through the gut-liver axis, triggering a cascade of immune responses aimed at preventing pathogen dissemination and maintaining hepatic function (Chaplin, 2010; Mogensen, 2009). In the liver, KCs serve as the first line of defense, and are adept at identifying and phagocytosing invading pathogens via pattern recognition receptors such as Toll-like receptors (TLRs). Moreover, they secrete cytokines and chemokines to recruit additional immune cells to the site of infection (Akira et al., 2006; Brubaker et al., 2015). Other innate immune cells, such as neutrophils, play key roles in the initial stages of immune responses by rapidly mobilizing, phagocytosing pathogens, releasing anti-microbial substances, forming neutrophil extracellular traps (NETs), and modulating other immune cells. Dendritic cells (DCs) capture and process antigens, presenting them to T cells to initiate adaptive immune responses (van der Windt et al., 2018).

The liver functions as a primary gateway between the intestine and systemic circulation, facilitating unidirectional blood flow from the portal vein to the central vein. This flow establishes distinct zones, including periportal (PV) and pericentral (CV) regions (Jungermann and Kietzmann, 1996; Mizukami et al., 2020). These zones exhibit differences in immune function (Papachristoforou and Ramachandran, 2022; Gola et al., 2021a). The PV zone’s immune milieu leans towards suppression, primarily influenced by elevated levels of immunosuppressive cells like IL-10 and Marco^+^ macrophages. These macrophages serve as frontline defenders, eliminating PAMPs and DAMPs to mitigate excessive inflammatory reactions. In contrast, the CV zone houses a higher density of cytotoxic T cells and other pro-inflammatory cells (Miyamoto et al., 2024). Macrophages within the central vein zone exhibit a heightened propensity towards pro-inflammatory reactions, are adept at swiftly addressing and eliminating potential pathogens. This robust immune response ensures pathogens are neutralized prior to their dissemination from the liver into systemic circulation.

Recent investigations employing spatial transcriptomics have revealed significant changes in the distribution and clustering patterns of neutrophils within regions of liver injury subsequent to EmKC depletion. Initially, neutrophils preferentially aggregate at the sites of injury in the CV zone, then disperse diffusely in the injured areas of both PV and CV zones. Additionally, a subset of gut microbiota-induced Marco^+^ immunosuppressive macrophages was detected in the PV zones by zone-specific scRNA-seq, and these macrophages were responsible for limiting excessive inflammation at the entry point of liver. Dysregulation of this self-regulatory system may promote the development of liver-related inflammatory diseases, such as MASH (Miyamoto et al., 2024). This study highlights the regulatory role of the gut microbiota in modulating the liver immune milieu and the progression of MASH.

### 2.6 Targeting macrophages for the treatment of MASH

Macrophages play a significant role in MASH, and the signaling pathways or molecules involved in recruitment, activation, and proliferation of macrophages may be potential targets for therapeutic intervention. Drugs such as Anti-CD163–IgG–dexamethasone (Svendsen et al., 2017), GR-MD-02 (Harrison et al., 2016), and Cenicriviroc (Friedman et al., 2018a) were designed directly targeting the macrophage to inhibit inflammatory responses. There are also indirect regulators of macrophages, such as Obeticholic Acid (Neuschwander-Tetri et al., 2015), Semaglutide (Cui et al., 2022), Elafibranor Acid (Ratziu et al., 2016), and Selonsertib (Loomba et al., 2018). Obeticholic Acid (OCA) has demonstrated significant anti-inflammatory and anti-fibrotic effects in animal models of MASH (Gan et al., 2014; Scott et al., 2018). Recent research has shown that OCA can directly inhibit the activation of NLRP3 inflammasome in macrophages, independent of FXR (Huang et al., 2021).

Improvement of MASH can also be achieved by modulating receptors on macrophages, such as vitamin D receptors (Dong et al., 2020) and MerTK (Cai et al., 2020). Currently, the kinase receptor-interacting protein 1 (RIP1) is a hot topic in various pathophysiological processes due to its regulation ability on cell death and inflammation. As research has shown, RIP1 is phosphorylated and activated primarily in liver macrophages (especially MoMF) during experimental and clinical MASH processes, leading to inflammasome activation and cell death (Tao et al., 2021). Since single-target drugs in clinical trials have shown limited efficacy against the complex mechanisms of MASH, recent research has proposed combination therapies, such as semaglutide with firsocostat and/or cilofexor. Furthermore, a novel multifunctional transcription factor, NRF2, has been identified. It regulates various homeostatic processes, including lipid metabolism, inflammation, and oxidative stress, highlighting its potential therapeutic value in MASH treatment (Fernández-Ginés et al., 2024). Overall, these studies reveal the specific roles and mechanisms of macrophages in MASH. By modulating KCs activation, inhibiting monocyte recruitment, and polarization, innovative KCs targeting strategies can be developed to mitigate liver inflammation and injury, and promote liver repair and regeneration.

## 3 Liver sinusoidal endothelial cells (LSECs)

### 3.1 Features of LSECs in health and MASH

Research has demonstrated that the metabolic functions of human liver cells are primarily regulated by non-parenchymal cells (NPCs), rather than the hepatocytes themselves (Kaffe et al., 2023). Liver sinusoidal endothelial cells (LSECs) constitute the largest cluster of NPCs population. Hepatic immune balance regulation mainly occurs within the liver sinusoids, therefore, the dysfunction of LSECs is considered a key factor in the occurrence and progression of MASH. Liver sinusoids are specialized vascular structures within hepatic lobules where blood flows from the portal vein and hepatic artery towards the central vein. LSECs line the sinusoids sparsely and possess high porosity (fenestration), allowing for the exchange of substances between blood and hepatocytes. This unique physiological feature renders LSECs the capability of regulating blood flow, facilitating metabolic exchange, immune tolerance, waste clearance, and modulating local inflammatory responses (Figure 1).

In fibrotic livers, extracellular matrix deposition occurs around the bile ducts and within the perisinusoidal space, leading to defenestration of LSECs. The loss of fenestration impairs the exchange of substances between hepatocytes and blood, resulting in metabolic disorders in liver cells (Gracia-Sancho et al., 2019). With the application of scRNA-seq, gene expression characteristics among LSEC-subpopulations have been well characterized, especially the specific functions. As reported in mouse cirrhosis model, the LSECs could be distinguished for 3 subpopulations with respective gene signatures and spatial localizations: The periportal LSECs CD36^high^, Mid-zone LSECs Lyve1^high^, and pericentral LSECs Kit^high^. Notably, pericentral LSECs, which also express CD34 (a potential marker) tend to be more vulnerable for capillarization (Su et al., 2021). In addition, a new marker for LSECs: Oit3, has been discovered due to its specific expression, may serve as a potential diagnostic marker for liver diseases, for functional changes in liver fibrosis (Li Z. W. et al., 2023). Some studies also indicate that LSECs injury could be a crucial “gatekeeper” in the progression from MASLD to MASH: LSECs injury precedes the activation of KCs and HSCs. Capillarization of LSECs occurs prior to MASLD progression (Miyao et al., 2015). Early intervention in LSECs injury may mitigate or delay the onset and progression of MASH. LSECs restoration not only aids in preventing MASLD progression but may also reverse existing tissue damage.

In short, while the precise mechanisms underlying LSECs capillarization remain incompletely elucidated, current research has revealed key facets (Table.2). Continued investigations will further enhance our comprehensive understanding of the molecular underpinnings governing LSECs capillarization.

**TABLE 2:** ScRNA-seq analysis of zonation markers of 2 cell types in the mouse liver under different conditions (Dobie et al., 2019; Su et al., 2021; Li Z. W. et al., 2023; Mederacke et al., 2013; Iwaisako et al., 2014).

Cell	Location	Different Zone	Cell Markers	Different Zone	Cell Markers
Hepatic stellate cells (HSCs)	Localized in the Space of Disse	quiescent HSCs (central vein)	Hhip, Aldh2, Dcdc2a, Ptprt, Fcna, Nt5e, Vipr1	CaHSC (activated HSCs)	Loxl1, Sox4, Podn, Adamtsl2, Rspo3, Spon2, Lpar1+, Col1a1hi, Col1a2hi, Col3a1hi, Actahi, Mmp2hi, Timp1hi, Loxhi, Loxl1hi
		quiescent HSCs (portal vein)	Ngfr, Vipr1, Itgb3, Igfbp3, Hspa1a, Ttyh1	PaHSC (activated HSCs)	Ngfr, Igfbp3, Tagln, Rgs4, Il34, Itgb3, Lpar1-, Col1a1low, Col1a2low, Col3a1low, Actalow, Mmp2low, Timp1low, Loxlow, Loxl1low
Liver sinusoidal endothelial cells (LSECs)	Localized in the hepatic sinusoids within the hepatic lobules	Endo-pp (periportal endothelial cells)	Dll4, Efnb2, Msr1, Ltbp4, Ntn4, Adam23,CD36	Endo-pp (periportal endothelial cells)	Ednrb, Jag1, Lrg1, Efnb1, Ltbp4, Adgrg6, Ly6a, Kitl, Ntn4, Dll4, Hes1, Msr1
		Endo-mid (mid-zonal)	Lyve1, Ctsl, Stab2	Endo-mid (mid-zonal)	Lyve1, Ctsl
		Endo-pc (pericentral endothelial cells)	Wnt2, Kit, Cdh13, Wnt9b, Rspo3, Thbd, Fabp4, H2-q2	Endo-pc (pericentral endothelial cells)	Wnt9b, Rspo3, Cdh13, Wnt2, Plpp1, Kit, Lrp6, Plxnc1, Bmp2

### 3.2 LSECs paracrine regulation of hepatocyte metabolism and fibrosis

In the liver, NPCs regulate the metabolic functions of hepatocytes through paracrine signaling mechanisms. Ligands within the paracrine and/or autocrine signaling network of LSECs originate from HSCs, LSECs, and cholangiocyte clusters (Xiong et al., 2019). WNT2, secreted by LSECs, regulates cholesterol uptake and bile acid binding in hepatocytes through its receptor FZD5 (Frizzled-5). This mechanism is specific to humans, highlighting the critical role of LSECs in maintaining liver metabolic homeostasis (Xiong et al., 2019; Kaffe et al., 2023). The fibrotic niche in the liver comprises a complex microenvironment involving various cell types and their interactions, mainly driven by which are predominantly performed by HSCs, macrophages, LSECs, and hepatocytes. These fibrotic niche cells are composed of a series of subclusters with specific markers, such as TREM2^+^CD9^+^ macrophages, ACKR1^+^ and PLVAP^+^ endothelial cells, and PDGFRα^+^ colla-gen-producing myofibroblasts (Ramachandran et al., 2019). Under physiological conditions, LSECs regulate hepatic vascular tone, blood flow, and the functions of hepatocytes and HSCs by secreting nitric oxide (NO) and endothelin-1 (ET-1). However, during liver fibrosis, decreased NO and excessive ET-1 promote and exacerbate the progression of liver fibrosis (Iwakiri and Kim, 2015; Francque et al., 2012). NO is primarily generated by endothelial nitric oxide synthase (eNOS). In mouse models of MASH that the LSEC-specific Notch signaling pathway activation is observed, mainly due to the reduction of eNOS activity and the dysregulation of multiple capillary markers. Mechanical stretch can also activate the Notch1 signaling pathway (Hilscher et al., 2019; Fang et al., 2022; Sun and Harris, 2020). In liver tissue samples from human MASH patients, Notch1 signaling pathway activation correlates with disease progression and prognosis, suggesting the therapeutic potential of targeting the Notch1 signaling pathway in MASH (Romeo, 2019). Potentially, targeting the Notch signaling pathway and enhancing eNOS expression may improve endothelial cell function and alleviate liver inflammation and fibrosis. Additionally, a newly discovered antagonistic interaction between GATA4 and MYC in endothelial cells has been identified as a pathway leading to liver fibrosis in mice and humans (Winkler et al., 2021). In summary: During liver fibrosis, the reciprocal interactions among multiple cell types and signaling pathways may serve as potential therapeutic targets to improve LSECs function, therefore alleviating liver inflammation and fibrosis.

### 3.3 Interactions between LSECs, macrophages/KCs, and HSCs

LSECs are key cells that maintain the immune homeostasis of liver sinusoids by secreting various signaling molecules. The interaction between LSECs, KCs, and HSCs could affect the liver immune environment and fibrosis progression. Unlike most other vascular environments, liver sinusoids have a slow blood flow and low shear stress, making it easier for leukocytes to contact and adhere to endothelial cells within them. Approximately 80% of leukocyte adhesion in the liver occurs within the sinusoids (Wong et al., 1997). When KCs are depleted, HSCs and LSECs become immediately activated. These activated cells produce monocyte chemotactic factors (such as CCL2 and CXCL1), which attract and promote the migration and settlement of circulating monocytes into the liver (Miyao et al., 2015). Activated LSECs express higher levels of adhesion molecules (such as VCAM-1, VAP-1, and ICAM-1) and endothelial migration receptors (such as CXCR4), which promote monocyte adhesion and transmigration through the endothelial cell layer into liver tissue (Lalor et al., 2007; Liaskou et al., 2011; Jung et al., 2007) (Figure 3). In particular, VCAM-1 promotes LSEC capillarization during liver injury processes (Guo et al., 2022). Once monocytes enter the liver, they begin to differentiate into Kupffer cell-like cells under the influence of the liver microenvironment, particularly due to the signaling molecules secreted by LSECs and HSCs (Bonnardel et al., 2019). The Notch signaling pathway plays an essential role in this process, especially the activation of recombination signal-binding protein-Jkappa (RBP-J) induced by Delta-like ligand 4 (DLL4), facilitating monocyte differentiation into KCs, contributing to immune surveillance and repair processes in the liver (Sakai et al., 2019; Borggrefe and Oswald, 2009). Activated LSECs also secrete pro-fibrotic molecules (such as TGF-β), and contribute to a reduced bioavailability of NO, which promotes HSC activation. Under normal conditions, LSECs depend on NO and exhibit anti-fibrotic effects, significantly contributing to maintaining HSC quiescence. However, capillarized LSECs lose this regulatory function (Xie et al., 2012; Deleve et al., 2008). LSECs can secrete vascular endothelial growth factor (VEGF) and Kruppel-like factor 2 (KLF2) to influence the status and function of HSCs and delay fibrosis progression (Deleve et al., 2008; Marrone et al., 2013). Conversely, bone morphogenetic protein 9 (BMP9) secreted by HSCs can prevent LSECs capillarization (Desroches-Castan et al., 2019).

### 3.4 The dynamic crosstalk between LSECs and immune cells

LSECs are central to liver immune responses due to their dynamic interactions with immune cells: they modulate immune cell activation and function through the secretion of signaling molecules, facilitate the recruitment of immune cells, and promote transendothelial migration via direct cell-cell interactions (Muller, 2016). When recruited to inflammatory sites, lymphocytes become activated and undergo transendothelial migration through interactions with LSECs, specifically between integrins on lymphocyte surfaces and adhesion molecules on LSECs (Liaskou et al., 2011; Shetty et al., 2018; Shetty et al., 2011; Bruns et al., 2015). In addition to their interactions with macrophages/KCs and neutrophils, LSECs also orchestrate vascular signaling cascades that facilitate lymphocyte recruitment within the liver (Chaudhry et al., 2019; Eksteen et al., 2004; Papaioannou et al., 2023). Lymphocytes are a heterogeneous group of cells, including T lymphocytes (such as CD4^+^ T cells and CD8^+^ T cells), B lymphocytes, innate-like lymphocytes (such as NK cells, NKT cells, γδ T cells, MAIT cells, ILCs, and iNKT cells). These lymphocytes encompass diverse subsets that secrete a range of cytokines, modulate HSC activity, and shape the local hepatic microenvironment (Heymann and Tacke, 2016; Togashi et al., 2019) (Figure 3). CD4^+^ T cells, commonly referred to as helper T cells, combat infections and diseases by regulating and activating other immune cells. While CD8^+^ T cells possess the ability to identify and eliminate abnormal cells, directly executing cytotoxic responses (Heymann and Tacke, 2016).

Physiologically, LSECs attenuate lymphocyte activation and help to sustain liver immune tolerance (Kubes and Jenne, 2018; Limmer et al., 2005; Diehl et al., 2008). LSECs were found to be involved in regulating the differentiation of naïve CD4^+^ T cells into Tregs by activating the TGF-β signaling pathway (Diehl et al., 2008; Carambia et al., 2013; Carambia et al., 2014). The pro-inflammatory cytokine secretion of mouse T helper 1 (Th1) and T helper 17 (Th17) effector CD4^+^ T cells could also be suppressed by LSECs via IL-10 and Programmed Death 1 (PD-1) signaling (Carambia et al., 2013). In addition, LSECs induce tolerance in CD8^+^ T cells by suppressing their activation and cytotoxic functions, which aids in safeguarding normal tissues from damage (Deleve et al., 2008).

### 3.5 LSECs perceive gut microbiota to influence immune responses

Researchers have recently revealed that gut microbiota plays pivotal in the pathogenesis of MASLD/MASH and the associated mechanisms involving LSECs. In the early stage of MASLD, disruption of intestinal barrier function allows microbial components such as pathogens and endotoxins to enter the bloodstream via the portal vein, a phenomenon known as ‘leaky gut’ (Figure 3). LSECs are directly exposed to blood from the portal vein, coming into direct contact with microbiota in the bloodstream and their metabolites such as lipopolysaccharides (LPS), forming a unique blood filtering barrier (Poisson et al., 2017; La Mura et al., 2014). LSECs exert dual functions in liver structural maintenance and MYD88-mediated micro-biota detection. They detect pathogen-associated molecular patterns (PAMPs) through pattern recognition receptors (PRRs), mainly the TLRs. TLR4 primarily recognizes LPS from Gram-negative bacteria, while TLR5 recognizes bacterial flagellin proteins. Upon sensing microbial stimuli, LSECs activate a series of downstream reactions via the MYD88/NF-κB signaling pathway (Zheng et al., 2021). For example, ectopic *Escherichia coli* in the liver could induce Endothelial-to-Mesenchymal Transition (EndMT) in LSECs through the TLR5/MYD88/TWIST1 pathway. This transition causes a loss of endothelial markers (such as E-cadherin) in LSECs and an increase in mesenchymal markers (such as α-SMA, N-cadherin), which promotes the transition of LSECs from an endothelial to a mesenchymal phenotype with fibrosis-related characteristics (Shen et al., 2023). This illustrates how gut bacteria, especially *Escherichia coli*, can worsen MASLD/MASH by altering the phenotype of LSECs.

Upon sensing microbial stimuli, LSECs secrete different chemokines de-pending on where they were located in liver, coordinating the positioning of immune cells in the tissue to optimize host defense responses (Su et al., 2021; Gola et al., 2021b). This chemokines secretion pattern contributes to the formation of functionally specialized immune environments in the liver, which renders immune cells the rapid and effective responsiveness during infection or inflammatory reactions (Gola et al., 2021b). In conclusion, LSECs regulate the localization and activity of surrounding immune cells, contribute to the formation of a functional immune environment in the liver. This optimization allows the liver to effectively engage immune cells in defense against various pathogens or inflammatory responses, ensuring organized and efficient immune defense mechanisms in the host.

### 3.6 LSECs exhibit distinct gene expression characteristics

After liver injury, the capillarization of LSECs is a complex process involving pro-inflammatory cytokines, oxidative stress, angiogenic factors, activation of HSCs, and metabolic abnormalities. In MASH, the restoration of capillarization in LSECs is regulated by multiple factors, including the role of nitric oxide (NO). NO, as a vasodilator molecule, when reduced in LSECs, may lead to increased vascular resistance and reduced blood flow within the microcirculation, thereby impacting liver function and metabolism (Lundberg et al., 2015). With the application of scRNA-seq, the subtypes and gene expression characteristics of LSECs have further been explored. It has been found that during liver fibrosis, while the proportions of LSECs subtypes may change, certain regional LSEC signature genes are conservative between control and cirrhotic groups (Table 2). These conserved signature genes are likely essential in maintaining the fundamental functions and structure of LSECs (Su et al., 2021). In short, while the precise mechanisms precipitating LSECs capillarization remain incompletely elucidated, current research has unveiled pivotal facets. Continued investigations will enhance our holistic comprehension of the molecular underpinnings governing LSECs capillarization. The persistent expression of these conserved signature genes illuminates adaptive and functionally protective mechanisms of LSECs amid liver fibrosis, presenting novel perspectives and avenues for comprehending and intervening in hepatic diseases moving forward.

## 4 Hepatic stellate cells (HSCs)

### 4.1 The techniques for detecting HSCs

ScRNA-seq and single-nucleus RNA sequencing (snRNA-seq) are both powerful techniques used to explore heterogeneity in the transcriptome of individual cells. While similar in principle, each technique has its own strengths that make it ideal for different applications. For scRNA-seq, it could sequence both cytoplasmic and nuclear transcripts, providing researchers with a more comprehensive view of the whole cell transcriptome with high throughput. However, there are still shortcomings of scRNA-seq, such as: it requires extensive preparation to ensure compatibility, cannot be used for frozen tissue, and the dissociation process may introduce biases due to cellular stress. For snRNA-seq, the notable advantages are: flexibility for fresh, frozen, or fixed tissue samples; less stress response of cells; and high effectiveness for analyzing complex cell types like kidney cells, heart cells, and neurons (Wu et al., 2019; Wolfien et al., 2020; Grindberg et al., 2013); The limitation of snRNA-seq is the inability to sequence the cytoplasmic RNA.

In liver tissue, choosing a proper strategy of sample processing is crucial for obtaining an expected result. Due to the dissociation-related cell perturbation, scRNA-seq is hardly to capture both human and mouse liver’s parenchymal cell fraction, but could provide high resolution for detecting the immune cells in liver like T cells, B cells, macrophages, Neutrophils, Mast cells and Dendritic cells, etc. While snRNA-seq could enable the characterization of interzonal hepatocytes, HSCs, cholangiocytes, mesenchymal cells and endothelial cells, etc., and even reveal the rare subtypes of parenchymal cells (Nault et al., 2021; Carlessi et al., 2023; Andrews et al., 2024; Payen et al., 2021; MacParland et al., 2018; Richter et al., 2021; Andrews et al., 2022) (Table 3). Regarding to HSCs, both scRNA-seq and snRNA-seq data have been reported. The distinctions of HSCs transcriptome profiling acquired from two techniques would be discussed hereafter.

**TABLE 3:** Difference and applicability of scRNA-seq and snRNA-seq (Ramachandran et al., 2020; Guilliams et al., 2022; Bakken et al., 2018).

Feature	ScRNA-seq	SnRNA-seq
Sample type	Whole cells from fresh liver	Nuclei from fresh/frozen liver
Tissue handling	Requires viable cell dissociation	Nucleus extraction
Transcript capture	Cytoplasmic and nuclear transcripts	Nuclear transcripts
Liver cell representation	High quality detection for Non parenchymal cells, immune cells in live	High quality detection for liver parenchymal cells like hepatocytes, hepatic stellate cells (HSCs), cholangiocytes etc.
Best for	Mild liver conditions, live tissue	Advanced liver disease, fibrotic/necrotic tissues
Limitation	Dissociation-related cell perturbation	Inability for sequencing cytoplasmic RNA

### 4.2 HSCs are drivers of liver fibrosis formation

In the liver, HSCs serve as the principal originators of extracellular matrix (ECM) production in MASH, acting as the predominant effector cells in the progression of liver fibrosis. When stimulated by inflammatory signals, HSCs transition from quiescent cells storing vitamin A to activated myofibroblast-like cells. These activated cells possess proliferative, contractile, inflammatory, and chemotactic properties, promoting the synthesis of ECM components as part of a wound healing or scar formation (Wells and Schwabe, 2015). The excessive accumulation of ECM components overwhelms the hepatic intrinsic degradation capacity, resulting in the occurrence and progression of fibrosis. Normal tissue is replaced by non-functional scar tissue, impacting the structure and function of the liver and ultimately culminating in liver failure (Friedman, 1993). Research indicates that in MASH, approximately 80%–95% of collagen synthesis is derived from HSCs differentiated myofibroblast-like cells (Mederacke et al., 2013). Consequently, the activation of HSCs is now widely recognized as a driving factor in the development of liver damage and fibrosis (Krenkel et al., 2019; Iwaisako et al., 2014). In recent years, scRNA-seq technology has provided new insights into cellular heterogeneity in liver fibrosis (Rosenthal et al., 2021; Dobie et al., 2019; Krenkel et al., 2019; Ramachandran et al., 2019; Kostallari et al., 2022). Studies have shown that in various fibrosis models in mice, including MASH, fibrosis is primarily composed of HSCs, with only a small proportion being portal fibroblast-like cells (Krenkel et al., 2019). In patients with liver fibrosis, research has revealed several pro-fibrotic cell populations, including HSCs, mesothelial/portal fibroblast-like cells, vascular smooth muscle cells, and scar-associated HSCs. Similar to mouse data, human HSCs are identified as the major pro-fibrotic cells, particularly in generating collagen-producing interstitial cells. Studies have also highlighted the significant role of HSCs characterized by high levels of PDGFRα expression in the fibrosis process (Ramachandran et al., 2019).

### 4.3 Cellular networks driving HSCs activation

Numerous cell types within the liver engage in intricate signaling networks to regulate the activation of HSCs and the fibrosis process in MASH. These cells include hepatocytes, KCs/macrophages, LSECs, various immune cells (such as natural killer cells, dendritic cells, T lymphocytes, B lymphocytes), and platelets, among others (Figure 3). The interactions of LSECs and HSCs impact the balance of liver microenvironment, which specifically represents as blood flow regulation, metabolic functions, responds to liver injury and inflammation. LSECs also participate in modulating HSCs activity and function by releasing specific cytokines and secretions, which induce the differentiation of HSCs to myofibroblasts (Pellicoro et al., 2014). Recent studies employing scRNA-seq technology have yielded a comprehensive analysis of discrete subtypes of major sinusoidal cell types (HSCs, KCs, MoMF, and LSECs). These investigations have documented the dynamic transformations and interactions of these cells in drug-induced liver injury and early liver fibrosis mouse models. They emphasize the unique gene expression networks involved in HSC activation and transdifferentiation, pointing out the significant role of the PLVAP protein in HSC activation (Xiong et al., 2019).

### 4.4 Complex ligand-receptor interactions among cells in MASH

The subcluster transcriptome analysis provides scRNA-seq another key advantage: the ligand-receptor interaction profiling among diverse cell populations. Since 2019, based on ligand-receptor database, analysis tools for predicting ligand-receptor links involved in intercellular interactions have been developed by many different research groups with respective computational methods. Some of these tools, like CellChat (R package designed for inference, analysis, and visualization of cell-cell communication from single-cell and spatially resolved transcriptomics by using a simplified mass-action-based model), CellPhoneDB (a publicly available repository of human curated receptors, ligands and their interactions paired with a tool to interrogate users’ single-cell transcriptomics data), NicheNet (an algorithm which uses human or mouse gene expression data of interacting cells as input and combines this with a prior model that integrates existing knowledge on ligand-to-target signaling paths), and SingleCellSignalR (a biology tool relying on comprehensive database of known ligand–receptor interactions to assist investigators in portraying cellular networks by inferring confident putative ligand–receptor interactions for follow-up validation), have already been widely applied in numerous biomedical studies (Cohen et al., 2018; Minutti et al., 2019; Vento-Tormo et al., 2018; Jin et al., 2021; Browaeys et al., 2020; Cabello-Aguilar et al., 2020; Efremova et al., 2020). Studies of ligand-receptor interactions between healthy and MASH mouse models have revealed that the HSCs, LSECs, and KCs/macrophages are prominent hubs for paracrine and autocrine signaling pathways, while hepatocytes are less involved (Xiong et al., 2019).

In the context of MASH, hepatocyte damage results in the release of various soluble mediators that signal surrounding cells of tissue injury. Surrounding cells perceive these danger signals (DAMPs) via pattern recognition receptors (PRRs). Upon stimulation by these danger signals, inflammasomes are assembled, activating pro-inflammatory cytokines such as IL-1β and IL-18 and initiating an inflammatory response (McHedlidze et al., 2013). For example, dead hepatocytes release P2Y14 ligands and nuclear protein high-mobility group box 1 (HMGB1), which can directly activate HSCs (Mederacke et al., 2022; Kao et al., 2008). IL-33 released by hepatocytes indirectly activates HSCs by recruiting ILC2, and also directly stimulate HSCs to produce ECM (Tan et al., 2018). Due to their high metabolic activity, hepatocytes contain abundant mitochondria. Mitochondria-derived DAMPs (mito-DAMPs) can induce HSC activation and contribute to liver fibrosis formation (An et al., 2020) (Figure 3).

In the MASH mouse model, different cell types within the liver, including scar-associated macrophages (SAMACs), endothelial cells (SAEndo cells), and mesenchymal cells (SAMes cells), exhibit significant paracrine and autocrine interactions (Ramachandran et al., 2019). SAMACs express various ligands, including AREG, IL-1β, TGF-β, TNFSF12, and PDGFB. These ligands bind to corresponding receptors on other cells, regulating cellular functions and the fibrosis process. SAEndo cells express high levels of Notch ligands JAG1, JAG2, and DLL4. The Notch signaling pathway plays a pivotal role in intercellular communication, particularly during liver fibrosis (Ramachandran et al., 2019). SAMes cells interact through receptors such as EGFR, IL1RA, TNFRSF12A, PDGFRα, and Notch receptor NOTCH3, promoting SAMes cell activation and proliferation, thereby driving the fibrotic process forward (Ramachandran et al., 2019; Chen et al., 2012; Makino et al., 2017; McKee et al., 2015; Wilhelm et al., 2016; Hellerbrand, 2013). HSCs serve as signaling hubs in the liver by secreting various factors termed “stellakines”. These “stellakines” primarily affect LSECs and immune cells. During MASH, an activated phenotype of HSCs may enhance autocrine IL-11 signaling, exacerbating liver fibrosis progression (Xiong et al., 2019). Receptors expressed on HSCs can be categorized into three main classes: (1) those related to ECM biology and fibrosis (e.g., Integrins and CD44 receptors); (2) those involved in extracellular factor signaling (e.g., Platelet-derived growth factor receptors/PDGFR and Insulin-like Growth Factor 1 Receptor/IGF-1R); and (3) vascular activity receptors (e.g., angiotensin II receptor type 1a/Agtr1a, endothelin receptor/Ednr, and Adrenergic receptor α2b/Adra2b) (Krenkel et al., 2019; Li Y. et al., 2023; Yokosaki and Nishimichi, 2021; Yasuda et al., 2009; Suzuki et al., 2023; Reynaert et al., 2008). It has been observed in both mice and humans that HSCs express receptors that promote contraction (Ednrb and Adrα2b) as well as GPCRs related to vasodilator peptides (Ramp1, *P*th1r, and Vipr1) (Xiong et al., 2019). These data underscore the critical role of HSCs in coordinating liver injury responses. By activating these receptors, HSCs can regulate their own contraction and proliferation states, further influencing hepatic vascular tone and fibrosis progression. In MASH, HSCs serve as integrators and transmitters of physiological signals to maintain liver sinusoidal homeostasis.

### 4.5 The fate of activated HSCs in MASH

Activated HSCs can undergo four possible fates, transformation into myofibroblast-like cells, transition to deactivated HSCs (dHSCs), inactivation, or entry into a senescent state (sHSCs). Activated HSCs (aHSCs) typically adopt a myofibroblast-like phenotype, secreting excessive extracellular matrix (ECM) components that lead to fibrosis, a primary cause of liver fibrosis. Some aHSCs revert to dHSCs (Kisseleva et al., 2012; Troeger et al., 2012; Nakano et al., 2020), returning to a dormant or quiescent state characterized by reduced ECM production, which slows or halts fibrosis progression. Other aHSCs may undergo inactivation, potentially resulting in cell death or permanent loss of function (Rosenthal et al., 2021; Zisser et al., 2021). Additionally, some aHSCs may enter a sHSCs. The exact role of senescent HSCs in fibrosis and liver cancer remains debated: some studies suggest that senescent HSCs in obesity-related liver cancer promote inflammation and tumorigenesis through the senescence-associated secretory phenotype (SASP). In contrast, others propose that aHSCs in a chronic liver injury environment can enter senescence, reduce collagen expression, and promote liver regeneration through SASP (Yoshimoto et al., 2013; Li et al., 2020; Miyazoe et al., 2020; Cheng et al., 2022; Lujambio et al., 2013). Recent studies have identified senescent HSCs in human and mouse MASH, highlighting potential markers such as MRC1, SLC9A9, PTPRB, STAB2, and SEMA6A through snRNA-seq rather than scRNA-seq (Yashaswini et al., 2024). These findings supply crucial insights for further exploring the role of senescent HSCs in MASH, particularly in targeted approaches to eliminate or modulate their effects.

### 4.6 HSCs during fibrosis regression

Preclinical research, clinical trials, and clinical observations provide evidence that liver fibrosis is reversible through various therapeutic approaches (Kisseleva and Brenner, 2021). Research shows that in MASLD, HSCs, as a primary source of hepatocyte growth factor (HGF), initially play a reparative role. The receptor for HGF, c-MET, is essential for hepatocyte regeneration and survival (Kroy et al., 2014; Huh et al., 2004; Giebeler et al., 2009). Moreover, HSC-derived collagen protects hepatocytes from various hepatotoxins and Fas-induced cell death (Bourbonnais et al., 2012). During fibrosis regression, HSC deactivation occurs through reversion to a quiescent phenotype or induction of apoptosis, leading to the elimination of activated HSCs (Kisseleva et al., 2012; Troeger et al., 2012). Furthermore, macrophages, key drivers of liver inflammation and fibrosis, can also transition to a reparative phenotype to support tissue regeneration. This transition can be induced by neutrophils or engulfment stimuli, resulting in increased secretion of matrix metalloproteinases (MMPs), which promote fibrosis reversal and liver regeneration (Calvente et al., 2019).

### 4.7 HSCs spatial zonation

The study of human HSCs heterogeneity was first reported by Adil El Taghdouini et al., in 2021 (Payen et al., 2021). They found that there are two subpopulations of human HSCs with unique gene expression signatures and distinct intralobular localization: the portal and central vein-concentrated GPC3^+^ HSCs and the perisinusoidally located DBH^+^ HSCs. The two kinds of HSCs, in addition to collaborating in the production and organization of the extracellular matrix, show distinct roles: GPC3^+^ HSCs are mainly responsible for glycosaminoglycan metabolism, while DBH^+^ HSCs display an antigen-presenting role by expressing related gene signatures (Payen et al., 2021).

Previous scRNA-seq studies have provided deeper insights into the heterogeneity and functional characteristics of HSCs across various liver diseases (Guerrero-Juarez et al., 2019; Peyser et al., 2019; Wells, 2014). However, reliable markers to distinguish different subtypes and states of HSCs remain lacking, posing challenges in studying fibrogenic HSCs in specific regions (Dobie et al., 2019). Excitingly, researchers have identified two distinct regions of HSCs using scRNA-seq: periportal-associated HSCs (PaHSCs) and central vein-associated HSCs (CaHSCs) (Mederacke et al., 2013). Table 2 lists the relevant regional markers. In the literature, CaHSCs are the predominant pathological collagen-producing cells induced by central lobular injury in liver fibrosis (Mederacke et al., 2013).

### 4.8 Drug development for MASH is challenging

Liver fibrosis is a major determinant of mortality in MASH (Diehl and Day, 2017). It remains a key focus in MASH research and a challenging target for drug development. Prolonged liver damage and inflammation lead to the replacement of liver cells by fibrotic scar tissue, significantly impairing liver function. Treatment options for advanced fibrosis are more limited compared to interventions targeting inflammation and lipid metabolism. Currently, there are no candidate drugs showing direct anti-fibrotic activity against HSCs in phase III clinical trials (Oseini and Sanyal, 2017). Recently, several studies have shown that maintaining HSCs in a quiescent state can effectively mitigate liver fibrosis (Tao et al., 2023; Bendixen et al., 2024). Besides the traditional liver pathways, extrahepatic pathways such as the gut-liver axis and thyroid receptor pathways also play critical roles in regulating MASH. Modulating gut microbiota balance through the gut-liver axis affects lipid absorption and inflammation levels, thereby improving liver health (Friedman et al., 2018b). Fortunately, the FDA has recently approved a drug for MASH treatment: Rezdiffra, a partial agonist of the thyroid hormone receptor. It improves liver function and structure by reducing fat accumulation in the liver through thyroid hormone modulation (Harrison et al., 2019). In conclusion, integrating therapeutic strategies targeting both intrahepatic and extrahepatic pathways could be a promising strategy for managing MASH and its related complications.

## 5 Conclusions and perspectives

MASLD/MASH presents substantial global challenges in terms of prevalence and drug development. Nevertheless, advancements in scRNA-seq technology have greatly deepened our understanding of the intricate pathophysiological mechanisms underlying MASLD/MASH. It has revealed intricate signaling networks and cellular interactions among liver cells, shedding light on diverse biological processes and cellular functions. These findings offer new perspectives on the cellular pathogenesis of MASLD/MASH, along with valuable insights for early disease diagnosis, the discovery of new biomarkers, the clarification of key mechanisms, and the identification of potential therapeutic targets. In the HSCs subset, researchers identified a uniquely expressed protein, LPAR-1, which could be targeted to inhibit liver fibrosis (Dobie et al., 2019). Scientists at the Icahn School of Medicine at Mount Sinai predicted an HSC-specific receptor-ligand autocrine loop mediated by neurotrophic receptor tyrosine kinase 3 (NTRK3) and neurotrophin 3 (NTF3) in both human and murine MASH, which could serve as novel candidate drug target for liver fibrosis (Wang et al., 2023). Additionally, in the MASH-associated macrophage population, scientists found that MS4A7 would be a potential target for alleviating liver injury (Zhou et al., 2024). Not only can scRNA-seq be used for mining new therapeutic targets, but it can also help identify non-invasive diagnostic signatures. Researchers developed a four-protein composite model (ADAMTSL2, AKR1B10, CFHR4, and TREM2) to evaluate at-risk steatohepatitis by deconvoluting scRNA-seq data (Govaere et al., 2023).

At the draw of liver scRNA-seq, scientists were not confine themselves in one technique means, multi-omics were well applicated. Combination of bulk RNA-seq and scRNA-seq could help to identify which cell type contributed the most of differentially expressed genes (DEGs) detected in whole liver tissue bulk RNA-seq. Further proteomics analyze could confirm that certain functional gene expressed in both transcriptional level and protein level (Xiong et al., 2019). Moreover, the integration of snRNA-seq and Single nucleus assay for transposase-accessible chromatin sequencing (snATAC-seq) could not only characterize hepatocytes trajectory in MASH progression but also reveal the TFs potentially responsible for this hepatocyte alteration (Xiao et al., 2023; Khanal et al., 2024). Disturbance of lipid metabolism plays a pivotal role in developing metabolic dysfunction-associated steatohepatitis (MASH), the application of Cryogenic dual-SIMS integrated metabolomics, lipidomics, and proteomics in the same liver lobules at single-cell level would make MASH pathogenesis mechanism research more elaborate and efficient (Tian et al., 2024). Even a powerful tool for transcriptomic study, scRNA-seq remains limitation and drawbacks like: high cost (specialized equipment, reagents, computational resources), data complexity (high noise levels, data sparsity) as well as what we discussed above the biological limitations (isolation disturbance, cell capture preference) and Limited Multi-Omic Integration (immature single cell proteomics and metabolomics) (Sun et al., 2021; Duhan et al., 2024; Fan et al., 2020; Wu et al., 2024). Nevertheless, with the continuous cost reduction, increasingly refined data processing interface, sophisticated database, and developed single cell multi-omics, scRNA-seq would become advanced, simple-to-use tool which is widely applied in biomedical research.
